# Remote Diffusion-Weighted Imaging Lesions in Intracerebral Hemorrhage: Characteristics, Mechanisms, Outcomes, and Therapeutic Implications

**DOI:** 10.3389/fneur.2017.00678

**Published:** 2017-12-15

**Authors:** Xu-hua Xu, Ting Gao, Wen-ji Zhang, Lu-sha Tong, Feng Gao

**Affiliations:** ^1^School of Medicine, Zhejiang University, Hangzhou, China; ^2^Department of Neurology, The Fourth Affiliated Hospital, School of Medicine, Zhejiang University, Yiwu, China; ^3^Department of Radiology, The Fourth Affiliated Hospital, School of Medicine, Zhejiang University, Yiwu, China; ^4^Department of Neurology, The Second Affiliated Hospital, School of Medicine, Zhejiang University, Hangzhou, China

**Keywords:** intracerebral hemorrhage, secondary brain injury, remote diffusion-weighed imaging lesions, microangiopathy, blood pressure lowering

## Abstract

Spontaneous intracerebral hemorrhage (ICH) is one of the most fatal form of stroke, with high mortality and disability rate. Small diffusion-weighed imaging lesions are not rare to see in regions remote from the hematoma after ICH and have been generally considered as related with poor outcome. In this review, we described the characteristics of remote ischemic lesions, discussed the possible mechanisms and clinical outcomes of these lesions, and evaluated the potential therapeutic implications.

## Introduction

Spontaneous intracerebral hemorrhage (ICH), defined as non-traumatic bleeding into the brain parenchyma (1), is the second most common cause of stroke (2), accounting for about 10–15% of all strokes worldwide each year (3), but 27–51% in Chinese population (4). Despite significant progress in clinical treatment, only 12–26% patients are able to lead independent lives at 12 months after ICH onset, and the mortality rate remains high, reported from 40 to 50% (5). It has been widely accepted that the hematoma could not only cause direct destruction of brain parenchymal tissue due to the mass effect known as primary injury (6) but also secondary brain injury, including perihematomal edema (PHE), secondary ischemic injury, and secondary micro-hemorrhage, each of which may lead to severe neurological deficits and sometimes delayed fatality (7, 8). Secondary ischemic lesions after ICH, both in perihematomal region (9–17) and remote region from the hematoma (9, 18–27) have been described recently. It is presumed that perihematomal lesion is related with decreased cerebral blood flow (CBF) around hematoma, and the mechanisms involved microvascular compromise, or bioenergetic compromise (9–17). While for remote site ischemic lesions, the underlying mechanisms are quite different. Diffusion-restricted lesions on diffusion-weighted imaging (DWI) are detected in 11.1–41% ICH patients remote from the hematoma (9, 18–27). Thus, they have been generally considered as cerebral infarcts. They are described as small, cortical, or subcortical, single or multiple, and topographically distant from the hematoma. They are often subclinical, but was reported associating with worsened outcome (20, 23, 24). Hence, it is important to understand the pathophysiology of these remote ischemic lesions after ICH for clinical practice. In this review, we first describe the characteristics and clinical outcomes of remote DWI lesions, then we discuss the possible mechanisms behind these lesions, which may provide clues for its potential therapeutic implications to ICH.

## Characteristics of Remote DWI Lesions

Acute ischemic lesions can be identified with high accuracy by using DWI technique (88–100% sensitivity and 95–100% specificity) (28). With DWI, lesions with restricted diffusion have been observed in 11.1–41% of patients with acute ICH (Table 1) (9, 18–27). These lesions are topographically distant from initial hematoma, and Gioia et al. found that more than half were in the hemisphere contralateral to the hematoma (9). They are mainly located in cortical or subcortical regions and can also be found in basal ganglia (BG), cerebellum, brain stem, or corpus callosum. These DWI lesions are mostly small, dot-like (<20 mm diameter or <0.5 mL volume), single, or multiple. Some patients are reported as having larger, territorial infarcts, while those patients usually received craniotomy or external ventricular drainage placement (18).

**Table 1:** Characteristics of remote DWI lesions.

Sample size	Design	ICH cause	Time of MRI	Prevalence	Location	Size	Number	Reference
78 (87% ICH)	Retrospective case-control	100% CAA	48.7% <7 days post-ICH	15%	64.7% subcortical	Not mention	1 or 2	Kimberly et al. (25)
					23.5% cortical			
					11.8% other			

118 (100% ICH)	Retrospective	70.3% HTN	1–3 days post-ICH	23%	70.4% subcortical	0.12–0.54 mL volume	Not mentioned	Prabhakaran et al. (27)
		11.9% CAA			25.9% cortical			
		13.6% anticoagulation			3.7% mixed			

114 (100% ICH)	Multicenter case-control	34% CAA	55% within 1 week	13%	75% cortical or subcortical	CAA 0.42–1.46 cm diameter	Not mentioned	Gregoire et al. (19)
		66% non-CAA		23% CAA	25% other	Non-CAA: 0.25–1.50 cm diameter		

95 (100% ICH)	Prospective	62% HTN	0.8–7.5 days	41%	Not mentioned	Not mentioned	Not mentioned	Garg et al. (23)
		15% CAA						

138 (100% ICH)	Prospective	62% HTN	Baseline: mean 2 days	Acute: 35% Follow-up: 27%	Baseline: 44% lobar	Baseline: mean 0.5 mL volume	Single or multiple	Menon et al. (26)
		1.5% CAA	Follow-up: mean 35 days		Follow-up: 13% lobar	1 month: 0.4 mL volume		

97 (100% ICH)	Prospective	100% HTN	Within 3 days	26.80%	75.5% subcortical or brainstem	<3 mm diameter	76.9% single	Kang et al. (24)
					24.5% cortical		23.1% multiple	

86 (100% ICH)	Prospective	Not mentioned	Within 14 days	17.40%	47% isolated cortical	86.7% small, dot-like	47% single	Arsava et al. (18)
					47% subcortical with or without cortical	13.3% larger, territorial	53% multiple	
					7% isolated deep			

153 (100% ICH)	Prospective	Mostly HTN	Baseline: within 2 weeks	11.1%	80% cortical or subcortical	Not mentioned	64.7% single	Tsai et al. (20)
			Follow-up: 3 months	52% not visible in follow-up	20% deep, cerebellum, brainstem			

392 (100% ICH)	Retrospective	15.2% CAA	Acute: ≤7 days	Acute: 18.2%	87.4% lober	Not mentioned	Not mentioned	Auriel et al. (21)
		17.2% HTN	Nonacute: ≥14 days	Nonacute: 12.9%				

117 (100% ICH)	Retrospective	45.7% HTN	1–5 days	14.50%	50% cortical	0.44 ± 0.3 mL volume	Not mentioned	Gioia et al. (9)
		9.4% CAA			47.6% subcortical			
		8.5% anticoagulation			2.4% infratentorial			
		55.2% HTN			46.4% cortical			

201 (100% ICH)	Retrospective	34.8% CAA	Within 1 month	27.90%	37.5% subcortical	<20 mm diameter	1–42 (median 2)	Wu et al. (22)
		10% Warfarin-related or undetermined						
					14.3% deep			
					1.8% cerebellum			

*DWI, diffusion-weighted imaging; ICH, intracerebral hemorrhage; MB, microbleeds; CAA, cerebral amyloid angiopathy; HTN, hypertension; BP, blood pressure*.

It is well known that spontaneous ICH was related with two different types of vascular disorders. Varied etiologies would lead to different imaging demonstration for ICH. One main etiology for ICH is cerebral amyloid angiopathy (CAA). It is characterized by the progressive accumulation of amyloid in the wall of small arteries and arterioles, which predominantly locate in the leptomeningeal space and cortex (29). Another cause of ICH is hypertension. Hypertension could modify arterial wall properties in another way and induce hyalinization and contribute to decreased elasticity of arteries, especially penetrating arteries located in the BG, thalamus, pons, and cerebellum (30). These pathological changes not only cause the vessel wall to become prone to rupture but also cause dysfunction of CBF regulation and luminal occlusion. Thus, CAA and hypertensive angiopathy (HA) ICH may both result in high rates of infarction. In a few studies, the DWI lesions were more commonly seen in patients with CAA-related ICH than in patients with HA-related ICH (19, 22). While in some other study, similar frequencies of DWI lesions were observed in both types of ICH (21). As reported earlier, there is a significant variation of hematoma locations of these two different etiologies. That is, CAA may be more prevalent in lobar ICH, and HA in deep ICH, which involves BG, thalamus, pons, and cerebellum (31). Despite of this, DWI lesions of these two types of ICH are still mainly located in cortical or subcortical regions, regardless of different etiology. According to the study conducted by Auriel et al., patients with CAA etiology may have relative higher occurrence of lesions in frontal lobe, and patients with HA in parietal lobe (21). The relative different distribution between the two may suggest yet unidentified processes contributing to the infarctions. Another explanation may be that the clinical distinction of CAA and HA is likely to be an oversimplification. In a research of postmortem brains of hypertensive patients with ICH, the authors found that even in deep ICH, CAA may still play a role in the pathogenesis of ICH (32). Hypertension may also be correlated to increased amounts of amyloid-β deposited in the brain of ApoE4 carriers (33). And in a histological study of brain tissue obtained during surgery, more than 30% of CAA-related ICH patients showed multiple microangiopathic change (34). Whether the CAA or HA are the underlying mechanism of these lesions need further study. Since studies using postmortem brains with DWI lesions is rather rare, animal experimental studies may give us more hints. Moreover, bio-markers of these two pathologies are waiting to be explored.

The accurate time point of DWI lesions occurrence is not clear. The DWI lesions are likely to happen along with the development of ICH. Kang et al. observed that the frequency of DWI lesions at initial observation was only 7.7%, whereas 5-day MRI imaging showed an increased frequency up to 25% (24). However, some studies suggested that this effect may only exist at the hyper-acute stage of ICH. In a study conducted by Auriel et al., no difference was shown in frequency of lesions on mean 2 days after ICH and after 14 days in MRI scans (21). In contrast, in a multicenter cross-sectional study, about one-third of DWI lesions were detected within 1 week, 82% were detected within 1 month, and the latest DWI lesion detected was at 64 days after ICH (19). These findings may indicate an ongoing pathologic process, even in subacute stage of ICH.

The research about the evolution of DWI lesions in ICH patients is rare. Tsai et al. reported about 52% DWI lesions disappeared on follow-up T2-weighted or FLAIR imaging at 3 months after ICH, when others were still present in MRI (20). The underlying mechanism about the regression of DWI lesions remains unknown. Clinical observations have reported complete early reversal of hyperintensities on DWI of those clinically diagnosed stroke patients (35, 36). In a few of recent research, DWI reversal is mainly associated with small infarct volume in patients with TIA and minor stroke (37, 38). Thus, it is hypothesized that regression of a DWI lesion after ICH may indicate minor vasculopathy or transient vascular occlusion with early reperfusion (20).

Despite of numerous studies about DWI lesions after ICH, it is, however, difficult to compare these studies, due to the difference in study design, patient population, time to MRI, and imaging methodology.

## Mechanisms of Remote DWI Lesions

There are several potential mechanisms of remote DWI lesions after ICH, including microangiopathy, cerebral atherosclerosis, blood pressure (BP) lowering, remote extension of hematoma, and venous drainage disorder. These mechanisms may interact with each other in complicated ways. For example, it is hypothesized that patients with a greater burden of small vessel disease are at increased risk for loss of cerebral autoregulation during the acute stage of ICH, and ischemia may be precipitated more easily by acute BP reductions in those patients (39). Besides, distal embolization after cerebral angiography and cardioembolic sources seem plausible mechanisms of DWI lesion development in some ICH patients (9). Although these mechanisms mainly work in acute stage of ICH, it is assumed that some of these mechanisms like microangiopathy keep on working even in subacute stage of ICH (19).

## Microangiopathy

Microangiopathy, also described as small vessel diseases (SVD), is a group of pathological processes affecting the small arteries, arterioles, venules, and capillaries of the brain. HA and CAA are considered as two major causes of SVD (40). Generally, the imaging demonstration of SVD mainly included cerebral microbleeds (CMBs), lacunar infarction, enlarged perivascular spaces (EPVS), and white matter hyperintensities (WMH). It was reported that DWI lesions were associated with CMBs (19, 20, 24–26), WMH (9, 19, 20, 24), and centrum semiovale (CSO)-EPVS (22). CMBs are regarded as small chronic brain hemorrhages, mostly caused by blood–brain barrier deficiency of the cerebral small vessels (41). It is already widely accepted that high incidence of CMB was related with high risk of spontaneous ICH (42), especially the CAA patients. The antemortem brain MRI-observed CMBs were found highly associated with cerebral microinfarcts (CMI) at autopsy in patients with neuropathologic evidence of CAA (43). For another major demonstrations SVD, the white matter lesions, it was usually regarded as evolving from a combination of demyelination, lacunar infarcts, and axonal loss, as well as venous congestion (44). Charidimou et al. have observed that different distribution of subcortical WMH on MRI might provide insights into different dominant arteriopathy (45). In the research, the prevalence of multiple subcortical spots was higher in the CAA compared to the HA group, and peri-BG WMH pattern was more common in the HA-ICH vs. the CAA-ICH group (45). Perivascular spaces (PVS), also called Virchow–Robin spaces, are extensions of the subarachnoid space that accompany penetrating vessels entering the brain parenchyma (46). EPVS were reported to be associated with age, lacunar stroke subtype, and white matter lesions in a prospective study (47). High degree of PVS in BG may represent HA etiology in spontaneous ICH patients, and high degree of CSO-PVS may represent CAA etiology in spontaneous ICH patients (47–50). As the data showed by Wu and his colleagues, high CSO-EPVS were significantly associated with small acute DWI lesions in ICH patients (22). These findings may imply that, an underlying SVD may play a role in the formation of DWI lesions. In fact, a new DWI lesion itself has also been assumed by some researchers as one of the neuroimaging markers for SVD (51).

## Cerebral Atherosclerosis

Cerebral atherosclerosis may account for almost one-third of ischemic strokes, and the underlying mechanisms include artery-to-artery embolism, hemodynamic compromise, local branch occlusion, or a combination of those conditions (52). In fact, intra- and extracranial atherosclerotic disease have also been detected in approximately one-fifth of patients with spontaneous ICH (53). It might be possible that cerebral atherosclerosis may contribute to the formation of DWI lesions after ICH through mechanisms mentioned before. By far, the research about the relationship between atherosclerosis and DWI lesions is rare currently, and the number of patients with cerebral angiograms performed in ICH is small. In one research, 21 patients (21.6%) had diagnostic cerebral angiograms performed, and 1 had arterial stenosis relevant to DWI lesions (24). While in another research, the authors found no relation between cerebral atherosclerosis and DWI lesions (26). Besides, it is noteworthy that the relationship between cerebral atherosclerosis and DWI lesions may be partly due to the coexistence of SVD, as SVD and cerebral atherosclerosis often share common vascular risk factors like hypertension, diabetes mellitus, hyperlipidemia, and smoking (54–56). In fact, it is difficult to distinguish the effects of cerebral atherosclerosis from SVD completely. Some studies have reported that distal single small subcortical infarction (SSSI), according to the lesion location in relation to the parent artery, is closely associated with lipohyalinosis, while proximal infarction seems to be related with atherosclerosis (57, 58). As similar size between SSSI and remote DWI lesions, further studies may divide these ischemic lesions into distal or proximal infarction according to the lesion location in relation to the parent artery, and investigate whether indicators for SVD and atherosclerosis differ according to lesion location.

## BP Lowering

Although high BP are reported to be associated with hematoma expansion and poor outcome (59), BP management in the acute stage of ICH remains an issue of debate. When BP is lowered aggressively, several large studies failed to reveal remarkable improvements in clinical outcome (60–63). It is argued that aggressive BP lowering may also increase the possibility of cerebral hypoperfusion, and thus induce cerebral ischemia, which outweighs the benefits (64).

Autoregulation of the cerebral circulation is the regulating mechanism that keeps CBF constant within wide limits of arterial pressure (65). In chronic hypertension, this homeostasis is shifted toward higher arterial pressures, which impairs the tolerance to acute decreases in arterial pressure, while improving the tolerance to acute increases in arterial pressure (65). Thus, in the setting of ICH, with intracranial pressure (ICP) elevation, the deficiency of CBF autoregulation is furtherly aggravated (65). Besides, within the cerebrovascular tree, distal arteries possess a substantially higher myogenic activity than proximal arteries, which could be more easily to be affected by chronic hypertension (44). Therefore, BP lowering might provoke cerebral ischemia in ICH patients with chronic hypertension, especially in the territory of distal arteries.

For decades, there are conflicting reports about the relationship between BP lowering and DWI lesions after ICH. Several studies found an association between the BP lowering and the DWI lesions after ICH (20, 23, 26, 27), while other studies failed to prove the association, including two recent studies (9, 22). This discrepancy may partly due to different study design, patient selection and MRI time. Besides, in these studies, BP values were observed at different time points (admission, lowest or highest values before MRI or some other time points) during the acute phase of ICH, and the patients were not treated with the same protocol. In addition, BP lowering in most studies may not reflect true BP control, as it was merely calculated by maximal SBP drop. Gioia et al. attempted to assess global BP control over 24 h by assessing the weighted average BP over 24 h and found no relationship between DWI lesions and control of BP over the initial 24 h (9). It is argued that prospective observational study and randomized clinical trials are needed to test the hypothesis whether and to what extent BP lowering may provoke DWI lesions. In fact, a multicenter randomized trial—Intracerebral Hemorrhage Acutely Decreasing Arterial Pressure trial II (ICH ADAPT II) has been ongoing to specifically assess the rate of ischemic lesion development in patients randomized to two different BP treatment strategies (66).

## Remote Extension of Hematoma

Recently, another etiology of ICH-related DWI lesions was proposed that hematoma may affect not only the primary site of ictus but also the remote regions, through PVS or perineurium possibly, according to the prior studies (67, 68). It was found that larger ICH volume has higher incidence of DWI lesions (22, 26). PVS, as mentioned before, are extensions of the subarachnoid space that accompany penetrating vessels when enter the brain parenchyma, and they serve as draining channels for the brain (46). PVS also collects and carries lymph from the brain parenchyma to the deep cervical lymph nodes (45). The perineurium represents a protective continuum with the pia-arachnoid in the central nervous system and surrounds nerve fascicles within the epineurium throughout the peripheral nervous system (69). A previous study in an ICH rat model has demonstrated that spot hemorrhagic lesions located in perihematomal tissues, which are usually named as ring hemorrhage for its ring shape surrounding the blood vessels, may be formed by blood overflow from hematomas along the PVS or the perineurium (68). In another ICH rat model, the formed blood elements (red blood cell) were observed to extend mainly into the PVS and perineurium in the perihematomal tissue and ipsilateral brain regions near the hematoma, and the soluble blood elements (bovine serum albumin) extended more extensively to almost all regions of the brain (44). In ICH patients, MRI with susceptibility weighted imaging (SWI) revealed that paramagnetic substances spread along the PVS or the perineurium and such distribution could cause the formation of CMBs (70, 71). These results indicated that blood constituents could extend through PVS and perineurium in ICH patients. Some reports support the notion that blood cell decomposition products such as iron, heme, and thrombin, as well as inflammatory cells, such as microglia and neutrophils, both contribute to the secondary perihematomal injury after ICH through various pathological pathways like cytotoxicity of blood, hypermetabolism, excitotoxicity, spreading depression, oxidative stress, and inflammation (8, 72). In ICH, inflammation can destroy the blood–brain barrier of small vessels by impairing endothelial function, which furtherly lead to microvasculature disruption and promote clot formation. If this effect sustained, irreversible ischemic injury would occur (73, 74). Hence, toxic substances extended through PVS and perineurium may also result in remote ischemic injury. In clinical practice, Wu et al. have found that high prevalence of CSO-EPVS were significantly associated with DWI lesions in ICH patients (22). Still, these findings cannot exclude the impact of potential microangiopathy and further study about the effect of global inflammation is needed.

In addition, He et al. observed that both formed and soluble blood elements are drained to bilateral deep cervical lymph nodes, and the PVS dilation was closely related to the PHE (67). Therefore, extension of the hematoma may not only cause remote DWI lesions but also lymphostatic encephalopathy and PHE (67).

## Venous Drainage Disorder

Zhang et al. has raised a new concept of Vascular Neural Network, which partially spawned from traditional Chinese medicine and pathophysiological understanding of stroke treatment strategies that arterial and venous blood flow needs to be in harmony (75). Recently, more and more studies revealed that venous system abnormality may play an important role in the process of cerebral ischemia. It is reported that in subarachnoid hemorrhage, when flow velocity in the basal vein was significantly elevated above normal values in the following day, patients were found without delayed ischemic neurological deficit, while when flow velocities in the basal vein were significantly below normal in the following day, patients were found to have permanent deficit (76, 77). In another study, prominent hypointense veins were seen on SWI at 4 days after acute infarction in the first and second medial gyrus (78).

The structures of cerebral veins are distinctly different from arteries. First, small veins and venules do not have encompassing smooth muscle cell, instead, a network of stellates or glia exist around venous walls as well as pericytes (79). Thus, venous cannot contract as strongly as arteries. Additionally, compared with arteries, cerebral veins possess no valves to prevent the backflow of venous blood (80, 81). Third, under the conditions of elevated ICP or regional brain edema, the thin walls of the venules and small veins would easily collapse since they can be compressed (81–83). In addition, leukocytes adhere to both arterial and venous endothelium in microcirculation, especially during the development of hypoxia, which could result in vascular occlusion, and venous system may be more vulnerable than arterial system for its slower blood flow (74, 84). An acute reduction of arterial flow at 20% may cause a mild and almost harmless episode of cerebral ischemia, but if the venous flow is decreased acutely by 20%, blood may be accumulated in the capillary system, leading to an increase in ICP, flow decrease, even venous infarction (81). Thus, it is presumed that the venous system may change passively along with increased cerebral blood volume, hypertension, and mechanical compression, which may influence circulation flow, and lead to cerebral ischemia.

In the setting of ICH, ICP is first increased by hematoma, and then the cytotoxic and vasogenic edema even accelerate the PHE. All these factors come to compress related venous structures and hence result in a vicious cycle. So far, few studies about the potential roles of venous side in DWI lesions after ICH have been reported. In future, both animal and human studies are needed to evaluate venous system disorder after ICH and its function in SBI including DWI lesions. Besides, imaging technologies detected small vein and venule also have to be developed.

## Clinical Outcomes

Most DWI lesions have been reported to be subclinical and did not lead to any neurologic deficit in the acute stage of ICH. Nonetheless, whether the presence of DWI lesions can predict poor functional outcome is still under debate. In a prospective study with 95 patients enrolled, DWI lesions were associated with nearly five times risk of dependence or death at 3 months after controlling other confounding factors (23). Kang et al. found that DWI lesions were an independent predictor of the composite of ischemic stroke and recurrent ICH, as well as vascular deaths during a median follow-up of 42 months (24). However, in another larger prospective study with 153 patients enrolled, the author failed to find relation between DWI lesions and functional outcomes at 3 months, and interestingly, they found the patients with more DWI lesions, which were not visible on follow-up T2WI or FLAIR images had favorable outcomes (20). As mentioned before, it was hypothesized that regression of a DWI lesion may indicate a minor vasculopathy and a transient vascular occlusion with early reperfusion (20).

However, these studies have certain limitations. For example, the time interval of a MRI scan after ICH varies, the frequency of MRI scan is only once or twice, the number of patients with DWI lesions is small, and observation period is different. Besides, cognitive outcomes are rarely reported among these studies. CMI is not only a major contributor to function disorder in humans but also an important cause of vascular or mixed dementia and gait disorder (85–87), and lesions on DWI have been supposed to represent acute CMI (88). In a prospective cohort study, a substantial risk of incident dementia (14.2%) was observed in dementia-free survivors of ICH at 1 year, and underlying CAA may be a contributing factor to the new occurrence (89). It was reported that detecting even a single DWI lesion may suggest a remarkable annual incidence of hundreds of new CMI (90). And a similar situation seems also exist in the patients with DWI lesions after ICH. In a prospective study, Menon et al. have found 87% of patients with DWI lesions at 1 month had one or more new DWI lesions compared to baseline, and 83% of all lesions at 1 month were new compared to baseline (26). As the cumulative effects of DWI lesions may lead to cognitive impairment, future clinical trials including patients with ICH should assess cognitive endpoints, which may need longer observation.

## Therapeutic Implication

The safety of aggressive BP lowering at early stage of ICH has been concerned for decades. The Intensive BP Reduction in Acute Cerebral Hemorrhage 2 (INTERACT2) trial reported no reduction in the rate of death or severe disability, although did find improved functional outcomes in those with more intensive BP control (61). The most recent Antihypertensive Treatment of Acute Cerebral Hemorrhage II trial also showed no difference in the rate of death or disability with intensive BP lowering compared to standard reduction, but a higher rate of serious and renal adverse events (62). These trials did not assess the small DWI lesions after ICH, which are usually silent, and do not cause significant neurological deficiency. Under the condition of remote extension of hematoma and venous drainage disorder secondary to ICH, BP lowering was proposed to be easier to trigger ischemic lesions, especially in the patients with microangiopathy and cerebral atherosclerosis. Further studies with serial MRI are necessary to definitively determine whether and to what extent BP reduction is causally related to DWI lesions after ICH, and whether DWI lesions may modify the effect of BP lowering on long-term outcomes after ICH. Antiplatelet therapy might slightly increase the incidence of reoccurrence of ICH (91). However, the risk of immediate and delayed ICH from initiating antiplatelet therapy after ICH must be weighed against the morbidity of thrombotic complications, especially in patients with DWI lesions, for their implying of stroke (IS or ICH)-prone state after ICH. And statin therapy would face the same problem. As patients with cerebral angiography and AF may confront a substantially heightened risk of DWI lesions after ICH, whether and when to apply anticoagulation therapy in these patients requires further study. Given the substantial important role of venous system, future clinical management of ICH should include the recirculation concept, which focuses on the harmony between arterial and venous systems. In a word, the etiology of DWI lesions varies, thus for every patient, treatment requires individual concerns.

## Summary

In conclusion, small DWI lesions are common in the regions remote from the hematoma in the acute phase of ICH, and the occurrence may be mainly in the subacute phase of ICH. These lesions have been generally considered as ischemic infarcts. Mechanisms of these lesions after ICH are still unclear. According to the recent publications, several factors may play potential roles, including microangiopathy, CAA, BP lowering, remote extension of hematoma, venous drainage disorder. These lesions are often subclinical, but may be associated with worsened long-term outcome. After all, the occurrence of DWI lesions may help to differentiate the stroke-prone patients after ICH, in which BP lowering and other treatment should be applied more cautiously and individually.

## Ethics Statement

The study protocol has been approved by the institutional Human Research Ethics Committee of the second affiliated hospital of Zhejiang university.

## Author Contributions

X-hX: bring up the idea and write the original review. TG: search for papers and help to write the original review. W-jZ: suggest some useful points about the structure of review. L-sT: help to revise the whole framework and polish the language. FG: help to revise the whole framework and polish the language.

## Conflict of Interest Statement

The authors declare that the research was conducted in the absence of any commercial or financial relationships that could be construed as a potential conflict of interest. The reviewer WK and handling editor declared their shared affiliation.
